# In-vitro study of Ketoprofen Release from Synthesized Silica Aerogels (as Drug Carriers) and Evaluation of Mathematical Kinetic Release Models

**Published:** 2018

**Authors:** Manijeh Mohammadian, Tahereh S. Jafarzadeh Kashi, Mohammad Erfan, Fatemeh Pashaei Soorbaghi

**Affiliations:** a *Department of Dental Biomaterials, School of Dentistry, Tehran University of Medical Sciences, Tehran, Iran. *; b *Iranian Tissue Bank and Research Center, Tehran University of Medical Sciences, Tehran, Iran.*; c *Department of Pharmaceutics, School of Pharmacy, Shahid Beheshti University of Medical Sciences, Tehran, Iran. *; d *Polymer Engineering Department, Faculty of Chemical Engineering, Tarbiat Modares University, Tehran, Iran.*

**Keywords:** Release kinetic model, Drug delivery, Silica aerogel, Ketoprofen, Drug dissolution

## Abstract

Silica aerogels are porous and extremely lightweight nano-materials that show interesting properties. These materials, because of biocompatibility, non-harmful to the body, and special physical characteristics such as large surface area and low density have great potential for use in a drug delivery system (DDS). The focus of this study is the evaluation of the effects of silica aerogels on improving the release rate of Ketoprofen as a relevant model drug of poorly soluble drugs in water. The in-vitro release rate of a conventional crystalline form of pure drug and three samples of drug loaded silica aerogels with different densities, 0.033, 0.080, and 0.24 g/cm^3^ were measured and investigated. The results show that all three samples of silica aerogels considerably increased (*p* < 0.05) the rate of drug release compared to its crystalline form. The silica aerogel sample with the lowest density (0.033 gr/cm^3^) has demonstrated the highest release rate of the drug (approximately five times faster than pure drug). Thus, silica aerogels could be acceptable carriers for poorly soluble drugs that require treatment with the fast release. Moreover, three release kinetic models were fitted with in-vitro drug release data and evaluated. The results indicate that the First-Order model is the best fit with the *in-vitro* Ketoprofen release data. Finally, in this article, a new kinetic release equation was obtained based on the first order model and release data, with applying the density of silica aerogel as an effective index parameter. This equation was proposed to describe Ketoprofen release rate in silica aerogels.

## Introduction

The pharmaceutical industry has a strong tendency to achieve a dosage form of the drug that has a desirable therapeutic effect with minimal side effects. Thus, scientists are trying to manufacture and develop pharmaceutical formulations that can reach the correct dose of the drug in a specific organ and their release will be reliable for a specified period of time (1, 2). In the past decades, it has become clear that the production and distribution of new drugs are solely not enough to warrant improvement in drug therapy (3). The development of appropriate drug carriers is beneficial to demonstrate existing problems in natural drug systems, such as quick metabolism, low absorption, deletion of the great variability of plasma, and low solubility in water (4). In this study, Ketoprofen was used as a drug model. It is an NSAID and a derivative of propionic acid which has been widely used for pain relief and inflammation reduction. Ketoprofen is clinically used for treatments of osteoarthritis and rheumatoid arthritis (5). Ketoprofen has a low solubility in water, short biological half-life (about 2-2.5 h), and potential to make severe injury in the stomach such as stimulating the stomach or bleeding after disposal in high concentrations. Therefore, it can be used as a good candidate for loading on drug carriers, particularly, in silica aerogels, for controlling the release rate and dosage (6). Some properties of Ketoprofen are presented in Table 1.

Drug delivery is a way to release the drug substance at certain points of the body to achieve the desired therapeutic effects (7). The materials used for DDS should have controllable characteristics such as non-toxicity, absorption, and dissolution profiles (8). Design and production of innovative and convenient drug delivery systems is a vitally important field of medicine. Nanotechnology and new materials have contributed significantly to the improvement of this field (9). Researchers have introduced controlled drug release systems to solve the problems and limitations of conventional pharmaceutical methods (8, 9).

The drug release rate from a carrier is a fundamental factor in drug delivery systems (DDS). Depending on the type of medication and treatment, different release rates may be considered. Formulation of a drug can be designed based on different methods to achieve the desired release rate (10). The dissolution rate of a drug on a carrier can be changed by various mechanisms such as modifying the content of filler, the amount of the drug, the carrier material, and changing the compaction force applied to the pellet formation (11). To obtain a proper drug release formulation, it is important to choose a carrier material with desired physicochemical characteristics (12). 

The carrier surface area is a substantial parameter in controlling the drug absorption and release rate. Aerogels have a very large specific surface area, thus, they can be used as a carrier to improve drug dissolution and absorption (10). The silica materials are the most biocompatible ones compared to other materials used in drug carrier systems (13). The chemical structures of silica aerogels are similar to Aerosil and their clinical properties are harmless to human body (14). Surface functionalization of carrier material by different methods provides a controlled drug release according to clinical needs (13, 15). Recently, silica aerogels have created new developments in the field of DDSs due to the high drug loading, controlled release, improved release properties, increased low soluble drugs bioavailability, and improved drug stability (16-18). It is reported that the drug loading on silica aerogel was done by adsorption from the supercritical CO_2_ method shows acceptable drug release properties (14, 19). Currently, various new materials with micro- or mesopores are being investigated for controlled drug release (20, 21), among them aerogels produced by the sol-gel method are highly regarded (22, 23). Veronovski *et al*. investigated the surface area and volume of the carrier as a key parameter to control drug release (23). 

The silica aerogels have a network of open pore structure. This feature has facilitated the transfer of vapors through the whole volume of the material. In theory, we are able to deposit any combination of materials with a minimum partial vapor pressure in a silica aerogel (18).

When a new formulation of the drug is developed, the study of the drug release rate and its solubility would be very important. Quantitative analysis of the release rate values obtained from the mathematical models can be an important step in describing the release kinetics and delivery of new drugs (24). Mathematical models of drug release help in examining and optimizing extreme changes in the process of drug release in accordance with the conditions of treatment. Three mathematical release models including Zero-order, First-order, and Higuchi models were evaluated in this work.

The Zero-order model describes the DDS in a way that the rate of drug dissolution is independent of initial drug concentration and is expressed as follows:

Q_t_ = Q_O_ + k_O_t                     Equ. 1 

Where, Q_t_ , Q_O_ , k_O_ , and t are the amount of drug release at time t , the initial amount of drug in solution (usually zero), Zero-order constant, and time, respectively.

This model can be used to characterize various types of modified DDS, such as low water-soluble drugs. These models are particularly important in the certain classes of drugs such as delivery of antibiotics, regulation of blood pressure, pain control, and anti-depressants (25, 26).

The First-order release kinetic model can be used to characterize the absorption and release rate of some medicines. It can demonstrate the release rate of pharmaceutical dosage forms in porous media (24, 26, 27). This model can be described by the mathematical expressions below:

M_t_ = M_O_ e^-k^_1_^t^

or 

In (M_t _) = In (M_O_) -k^1^t                             Equ. 2

Q_t _= Q_O_(1- e^-k^_1_^t ^)                               Equ. 3

Where,

M_O_ = the initial amount of drug

M_t_ = the amount of drug remaining at time t

Q_O_ = the initial amount of drug

Q_t_ = the amount of drug release at time t

k_1_ = the first order model constant

Higuchi relation has been initially proposed to characterize the drug dissolution from matrix systems. Subsequently, this model has been used to define the drug release of the porous systems and other geometries. Application of Higuchi dissolution model for drugs with the modified release such as transdermal systems and matrix tablets has been reported in the literature. In this model, drug release is limited by penetration of solutes into the control matrix. Moreover, the release mechanisms follow the diffusion (25, 26, 28). The equation of this model is:

Q_t_ = k_h_ t                              Equ. 4

Where, Q_t_ is the amount of drug release at time t and k_h_ is the Higuchi model constant.

The aim of this paper is evaluation of the drug release from synthesized silica aerogels with different physical properties and loaded by Ketoprofen. Also, various mathematical models were examined to characterize the appropriate model describing the drug release kinetics in silica aerogels as DDSs.

We previously investigated the synthesis of silica aerogels with different physical properties and characterizing of their physicochemical structure and morphology by BET analysis, FTIR, XRD, FESEM, and TEM. Moreover, we evaluated the Ketoprofen loading of synthesized silica aerogels with supercritical CO_2_ method, cell culture, and cell viability of these materials (29). In this study, Ketoprofen release from silica aerogels was investigated. 

## Experimental

*Fabrication of silica aerogel*

The two-step sol-gel method was used to the fabrication of silica aerogel samples. A solution mixture of water, hydrochloric acid, and methanol with certain molar ratios was prepared. Tetra-methyl orthosilicate (TMOS) was added to the solution mixture and stirred in a stirrer for 30 min. When the mixing of material within solution was completed, an extra amount of ammonia and water solution was added to the mixture and three samples of the final mixture were prepared. Then, each sample was diluted by a certain concentration of acetonitrile to obtain the silica aerogel with targeted density. The silica gel samples were filled into the cylindrical molds and aged about three days at room temperature. Finally, the specimens were placed in the autoclave and dried by the supercritical drying technique at 40 °C with a pressure of 100 bars. We have described the details of the synthesis of silica aerogels in the previous work (29).

*Measuring of silica aerogel properties*

*Density of silica aerogel*

The bulk density of any produced silica aerogel was determined by measuring the mass and volume of a silica aerogel specimen. Each sample was weighted by a digital scale with a precision of 0.0001 grams and the volume of aerogels specimens were determined by the image processing method. To this end, a high-resolution camera (15 mega pixel) was fixed at a vertical distance of 15 cm from the specimens. Six images of each sample were taken from its 6 different faces by this fixed camera. Then, the images of aerogel samples were dimensional analyzed by the computer and hence the dimensions and volume of them were determined.

*BET analysis*

The standard BET (Brunauer–Emmett–Teller) analysis was used to determine the surface area and pore diameter of silica aerogel samples. The samples were degassed and taken under the adsorption and desorption with N_2_ gas inside the BET device. The pore diameters and surface areas were described by nitrogen adsorption isotherms obtained from BET tests. 

*Ketoprofen as model drug*

Ketoprofen was used as a model drug to study the release rate from drug loaded silica aerogels (new drug carrier systems). 

In this study, the supercritical CO_2_ method (as described in (29) was used to drug loading in aerogels. To perform this test, a certain amount of aerogel specimens and model drug (Ketoprofen) were weighed and placed inside the supercritical device at T = 40 °C. Then, CO_2_ was pumped to supercritical devices until the targeted pressure (*P* = 120 bars) was reached. The system was kept in this condition (T = 40 °C and *P* = 120 bars) for three days. 

*In-vitro* *evaluation of drug release from silica aerogels*

The results and recommendations which have been presented in literature for drug dissolution tests were used for measurement and evaluation of the drug release kinetics (30). Therefore, the 0.1N HCl solution was chosen as a medium solution. The USP dissolution apparatus with a basket was used to perform drug release test. The medium temperature was fixed at 37 °C.

First, a certain amount of pure Ketoprofen (14 mg) or powder of Ketoprofen loaded silica aerogel sample (50-80 mg) was weighed. To control of sink conditions, the value of drug taken in such a way that the final concentration of it in the medium solution was approximately 10% of the maximum solubility of Ketoprofen in 0.1N HCl. The sample was placed in the basket. Then, the basket was submerged inside the container containing 900 mL of 0.1N HCl at 37 °C and a mixer with speed of 100 RPM stirred the solution. At specified time intervals (5, 10, 15, 20, 30, 40, 50, 60, 100, 150,200, and 250 min for pure ketoprofen and 2, 5, 10, 15, 20, 25, 30, 40, 50, 60, 90, 120, 150, and 180 min for drug loaded silica aerogels) a sampling of dissolution was carried out with amounts of 2 mL. After each sampling 0.1N HCl solution was added into the container to keep the total volume of solution constant. The solution samples were filtered by a Nylon filter with micron size (0.45 μm). The absorbance of samples was read with a UV spectrophotometer at a wavelength of 259 nm. A standard curve was plotted for this purpose and used to determination of drug concentrations. Finally, the release rate of drug versus time was determined. The validation method of this UV spectroscopy procedure for measuring of drug concentration was presented in the next section.

**Table 1:** Properties of Ketoprofen

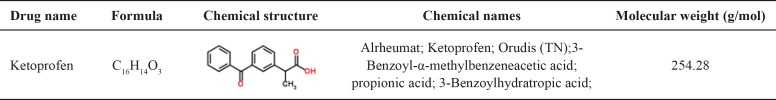

**Table 2:** Physical characteristics of used silica aerogels and amount of drug loading on them (data are expressed as mean ± SD, n = 3.)

**Sample**	**Final aerogel density** **(g/cm** ^3^ **)**	**BET surface area** **(m** ^2^ **/g)**	**BET average pore size (nm)**	**Loading efficiency (%)**
1	0.033	568.90±26	24.44±1.3	15.6±1.5
2	0.080	856.61±38	16.96±1.2	22.8±0.9
3	0.240	895.40±35	9.53±0.8	23.5±1.1

**Table 3:** The UV-Spectroscopy validation parameters for Ketoprofen (The dissolution medium was 0.1N HCl at 37 °C and data are expressed as mean ± SD).

**Validation Parameter**		**Value**	**acceptable specificity**
Calibration range $\left(\frac{\mathrm{\mu g}}{\mathrm{ml}}\right)$		1.6 -16	-
Calibration points		5	> 3
Correlation coefficient $\left(R^{2}\right)$		0.9996±0.0002	-
Slope		0.1001±0.0070	-
Intercept		0.0211±0.0025	-
LOD $\left(\frac{\mathrm{\mu g}}{\mathrm{ml}}\right)$		0.084	-
LOQ $\left(\frac{\mathrm{\mu g}}{\mathrm{ml}}\right)$		0.255	-
Precision (intra-day n=3) RSD%		0.480±0.045	< 5%
Precision (inter-day n=9) RSD%		0.0507±0.0931	< 5%
Accuracy (%Recovery)	Level 1 $\left(C=5\frac{\mathrm{\mu g}}{\mathrm{ml}}\right)$	100.14±2.71	> 95%
	Level 2$\left(C=10\frac{\mathrm{\mu g}}{\mathrm{ml}}\right)$	101.33±2.08	> 95%
	Level 3$\left(C=15\frac{\mathrm{\mu g}}{\mathrm{ml}}\right)$	99.47±1.20	> 95%

**Table 4:** The mathematical models data fit information

**Sample**	**Zero-order**		**First-order**		**Higuchi**	
	$k_{0}$	$R^{2}$	$k_{1}$	$R^{2}$	$k_{h}$	$R^{2}$
Pure Ketoprofen	0.3642	0.934	0.0086	0.970	5.4397	0.955
Silica aerogel (0.033gcm3)	0.4381	0.444	0.0708	0.995	11.011	0.552
Silica aerogel (0.08gcm3)	0.4789	0.568	0.0501	0.990	10.3949	0.733
Silica aerogel (0.24gcm3)	0.5026	0.642	0.0341	0.992	9.3498	0.853

**Figure 1:**
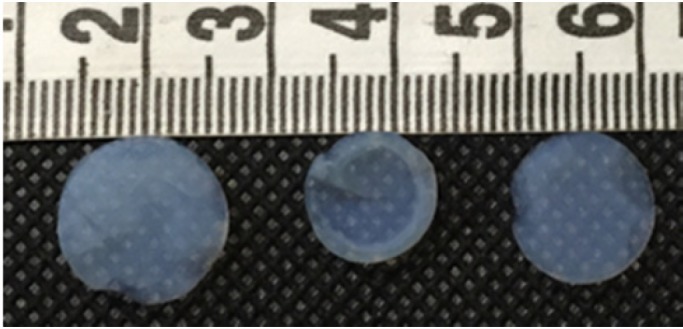
Image of silica aerogel samples.

**Figure 2:**
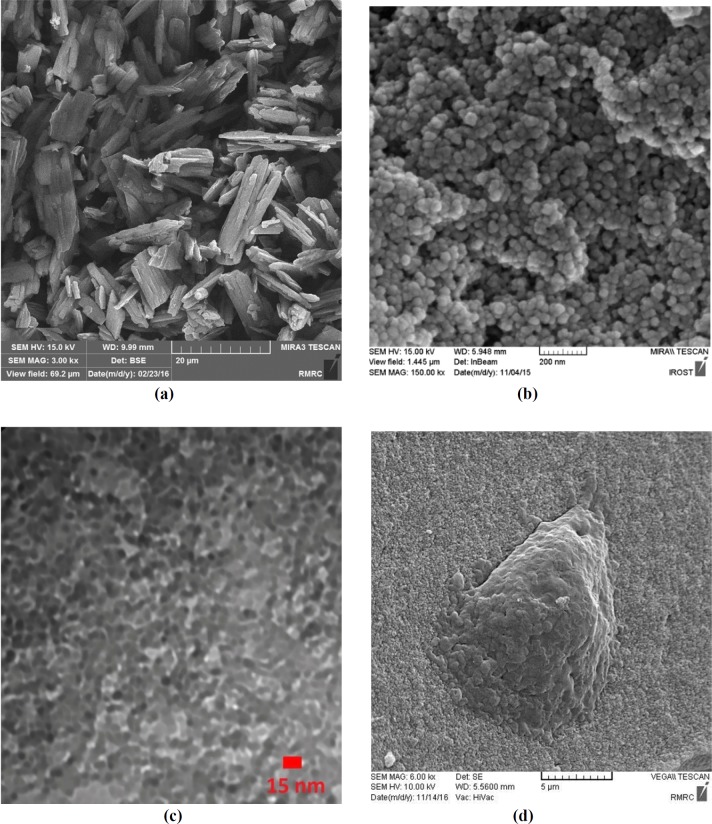
**(a)** FE-SEM image of pure Ketoprofen; (b**)** FE-SEM image of silica aerogel sample; c) TEM image of silica aerogel sample; (d**)** SEM cell growth morphology of silica aerogel.

**Figure 3:**
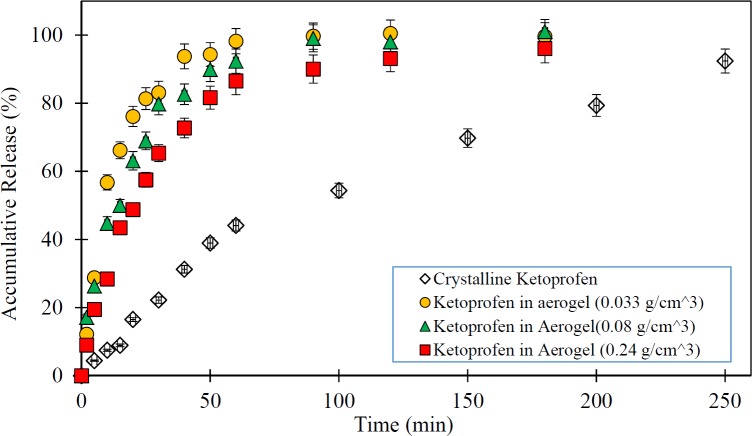
Release rate of pure Ketoprofen and drug loaded silica aerogels (The dissolution medium was 0.1N HCl at 37 °C and data are expressed as mean ± SD).

**Figure 4:**
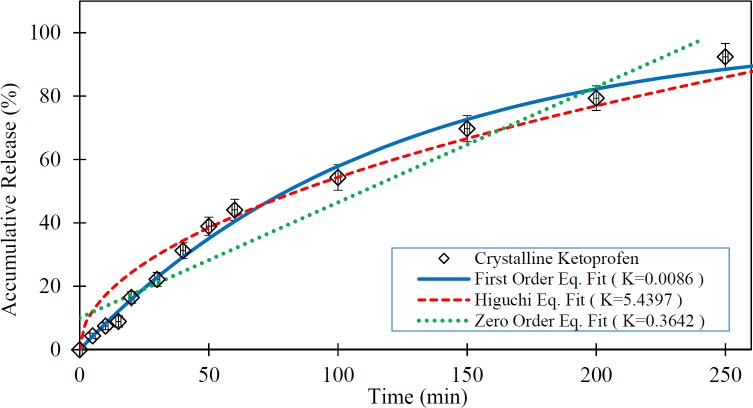
Release rate of pure Ketoprofen and fitted kinetic models (The dissolution medium was 0.1N HCl at 37 °C and data are expressed as mean ± SD).

**Figure 5:**
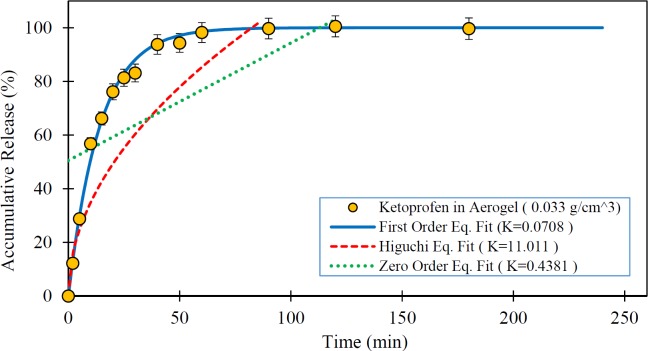
Release rate of Ketoprofen loaded in the silica aerogel ( ) and fitted kinetic models (The dissolution medium was 0.1N HCl at 37 °C and data are expressed as mean ± SD).

**Figure 6:**
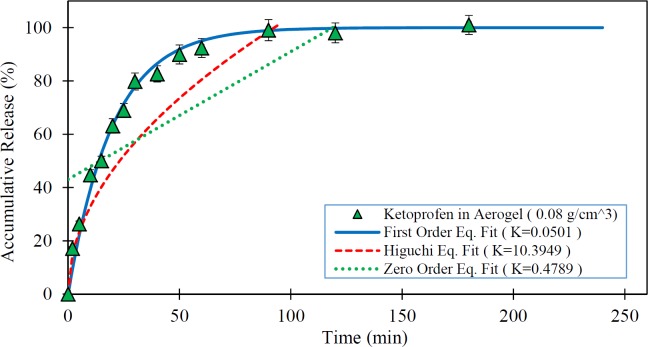
Release rate of Ketoprofen loaded in the silica aerogel ( ) and fitted kinetic models (The dissolution medium was 0.1N HCl at 37 °C and data are expressed as mean ± SD).

**Figure 7:**
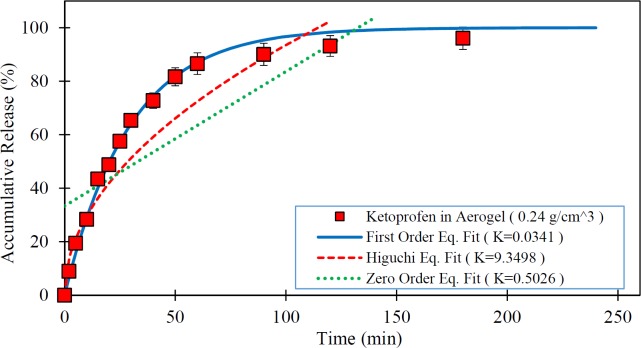
Release rate of Ketoprofen loaded in the silica aerogel ( ) and fitted kinetic models (The dissolution medium was 0.1N HCl at 37 °C and data are expressed as mean ± SD).

*Analytical method validation of UV-spectroscopy*

The UV spectroscopy was used to Ketoprofen in a 0.1N HCl solution at wavelengths range from 200 to 400 nm and a maximum wavelength of 259 nm was obtained for ketoprofen in this solution. The calibration curve was prepared in this maximum absorption wavelength (259 nm).

Then, this method was used to determination of Ketoprofen release concentrations. It is necessary to validate this method for pharmaceutical applications. Thus, this method was validated for linearity, inter-day, and intra-day precision, accuracy, stability, limit of detection (LOD) and limit of quantification (LOQ) in accordance with the ICH guidelines (31).

The linearity of Ketoprofen concentration in medium solution (0.1N HCl) with respect to UV-absorbance was evaluated by the construction of calibration curves corresponding to peak areas of 5 drug concentrations in range 1.6 to 16 μg/mL with 3 replicates by linear regression.

For assessment of intra-day precision (repeatability), the relative standard deviation percent (%RSD) was calculated for 3 replicates of quality control samples on the one day. Inter-day precision (intermediate precision) was estimated by calculation of the %RSD of these 3 samples in 3 replicates over 3 days.

The accuracy of the analytical method was verified by performing recovery study according to ICH guideline at three different concentrations levels 80%, 100%, 120%. For this purpose, the difference between conventional true value and measured concentration of 3 replicates for quality control samples is indicated as mentioned above.

The LOD and LOQ were estimated directly from the 3 calibration curves. LOD and LOQ were calculated as 3.3σ/S and 10σ/S, respectively, where σ is the standard deviation of intercept and S is the slope of the calibration curves.

*Investigation of drug release kinetics using mathematical models*

In this work, three release kinetics models (the Zero-order, First-order, and Higuchi models) were investigated to determine drug (Ketoprofen) release kinetics of silica aerogel as a DDS. First, the *in-vitr*o data of drug release were fitted to these models. Then, the kinetic models were evaluated based on the correlation coefficient. Finally, the best release kinetics model for Ketoprofen loaded silica aerogels was proposed.

*Statistical analysis*

All data and the quantitative results were expressed as mean ± SD of 3 replicates experimental data. The one-way analysis of variance (ANOVA) followed by the Tukey post-test is used to determination of any statistically significant differences between the means of data. A p-value less than 0.05 were considered statistically significant.

## Results and Discussion

*Characteristics of drug and silica aerogels*

In this study, three hydrophilic silica aerogels with different densities were synthesized and used as a drug (Ketoprofen) carrier. The silica aerogel samples were loaded with Ketoprofen as a drug model by the supercritical CO_2_ procedure. The physical characteristics of silica aerogels (surface area and pore diameter) have been extracted by BET analysis. Because the surface area and pore diameter of the silica aerogels have been a function of their densities, the density of silica aerogel was chosen as a physical characteristic index for investigation of its effects on the rate of drug release.

Figure 1 shows a sample image of produced silica aerogels. The physical characteristics of the silica aerogels and their drug loading efficiencies are shown in Table 2. Figure 2 shows the FE-SEM image of pure Ketoprofen. This Figure clearly shows the crystalline form of ketoprofen. 

Field Emission Scanning Electron Microscopy (FESEM), Transmission Electron Microscope (TEM) analysis, cell growth, and Scanning Electron Microscopy (SEM) imaging techniques were used to morphology evaluation of synthesized silica aerogels samples. The results have shown that the used silica aerogels have nano peruse networks and high potential in cell growth and cell survival. These materials are harmless to the human body as a drug carrier (29). Figure 2a shows the FE-SEM image of pure Ketoprofen and Figures 2b to 2d show the FE-SEM image, TEM image, and SEM cell growth morphology of a silica aerogel sample.

*The UV-spectroscopy validation*

The obtained validation parameters of the UV-Spectroscopy method including linearity, LOD and LOQ, intra-day and inter-day precisions, and accuracy of Ketoprofen in 0.1N HCl solution are presented in Table 3. The results show that the %RSD values of all concentrations were less than 5% hence, this method had an acceptable precision (31). Moreover, it is revealed that the accuracy was appropriate (32).

After the verification of the UV spectroscopic analysis method, a dissolution test was carried out to illustrate the possibility to apply this method to the quantitative analysis of the dissolved Ketoprofen in the dissolution media.

*In-vitro drug release from silica aerogels*

In this study, the impact of the physical characteristics of the drug-silica aerogel formulation on the rate of drug release was investigated. The low-water-soluble drugs such as ketoprofen have been problematic for drug delivery particularly for oral administration due to their slow release characteristics. Thus, the improvement of the dissolution rate of the drug can increase its bioavailability. Therefore, the silica aerogels potentials taking into account their different physical properties (density as index parameter) were assessed as a drug carrier in Ketoprofen release improvement. Figure 3 shows the release rate of pure Ketoprofen and drug loaded silica aerogels with different densities.

As shown in Figure 3 pure Ketoprofen has a very slow release. It is seen that in the first 50 min only about 40% of the pure Ketoprofen has been released and it took about 250 min to release nearly 90% of it. However, it is observed that all drug (Ketoprofen) loaded silica aerogels have a rapid release rate relative to the pure drug (*p* < 0.05) so that in the first 20 min more than 50% of the drug was released. The results illustrate that the release speed of the drug varies with the change of silica aerogel density (*p *< 0.05). Among the studied samples, the silica aerogel sample with a density of 0.033 g/m^3^ has been the one with the highest rate of drug release (*p *< 0.05). In this case, it releases nearly 100% of the drug in the first 50 min. By increasing the density of silica aerogels, their drug release rate was reduced. Indeed, in the case of silica aerogel sample with a density of 0.24 g/m^3 ^, the drug release rate was reduced to 80% in the first 50 min. This is due to the reduction in the diameter of the aerogel pores at high densities.

As presented in Table 2, the BET analysis results have shown that by increasing the aerogel density, the surface area of silica aerogel was increased and pore diameter size of it decreased. This is explained by compaction of the silica aerogels in high densities. Thus, based on the results with an increase in the pore size the release rate was increased (*p *< 0.05). The issue can be very important for the use of a silica aerogel as a drug carrier where its surface area and porosity have leading role in aerogel drug loading and release. Therefore, in medical applications and delivery systems production and use of silica aerogels with desirable properties based on medical and drug targets can be suitable.

*Evaluation of drug release kinetics using mathematical models*

In this study, the ability of three predefined mathematical models was investigated to describe the dissolution of the pure Ketoprofen and Ketoprofen from drug cattier systems (silica aerogels), using *in-vitro* data. For this objective, the drug release data obtained from tests were fitted to release kinetics mathematical relations. The *in-vitro* drug release rate of the pure Ketoprofen and the fitted mathematical models on in-vitro data are presented in Figure 4. As is clear from this Figure, the first order model shows the best goodness of fit. In this model the R-squared was obtained 0.992.

Figures 5 to 7 show the *in-vitro* drug release from silica aerogels with the density of 0.033, 0.08, and 0.24 grams per cubic centimeter and fitted mathematical models.

Table 4 shows the mathematical models data fit constants and the R-Squared for pure Ketoprofen and silica aerogels with different densities. 

As seen in the Figures 4 to 7 and Table 4, the first-order mathematical model illustrates a very good match with drug release from different silica aerogels and pure Ketoprofen. The correlation coefficient (R-Squared) of this model was high (> 0.97) in all samples. Therefore, it could well describe the kinematics of drug (Ketoprofen) release from the pharmaceutical carriers. Although, the Higuchi and Zero-order models approximately described the release rate of pure Ketoprofen with R^2^=0.955 and R^2^=0.934, but the results show that the zero-order and Higuchi models have the low correlation data coefficient (R-Squared) and hence these models are not able to properly describe the Ketoprofen release from silica aerogels (R^2 ^= 0.444 -0.642 for Zero-order model and R^2 ^= 0.552 -0.853 for Higuchi model).

With correlation of the experimental release data, the relationship between the density of silica aerogels and the first-order model constant was obtained as follows:

k_1_ = 0.0201 ρ^-0.367^

R^2 ^= 0.99                              Equ. 5

Where k_1_, and ρ are the first order model constant, and silica aerogel density, respectively.

According to the results of this research, the following equation is derived to describe the release kinetics of Ketoprofen loaded silica aerogels: 

Q_t_ = Q_O_(1- e^-(0.0201 ρ^^-0.367)^^t ^)                               Equ.6

This equation can predict the release rate of Ketoprofen from an aerogel with the known density. It can be very efficient than First-order model for drug release from silica aerogels due to consideration of their density variation. The developed model can help easily examine the changes influencing the drug release and hence the drug release was optimized in accordance with the conditions of treatment. The results showed that the use of mathematical models could be useful for studying the mechanism of release from a DDS. 

## Conclusion

In this work, drug release of hydrophilic silica aerogels (as drug carriers) with various densities and pure drug were investigated. The pore diameter and surface area of silica aerogels are functions of their densities. Thus, the density of aerogel was selected as a characteristic index and studied its impact on the drug release rate. Moreover, three release kinetics models include Zero-order, First-order, and Higuchi were evaluated to describe the release kinetics of the pure Ketoprofen and drug-loaded silica aerogels. The *in-vitro* drug release data showed that the Ketoprofen release rate of silica aerogels was much faster than that of the pure Ketoprofen. Therefore, in cases that a drug application is limited due to complications caused by the long-term administration the hydrophilic silica aerogels with a rapid drug release can be a suitable candidate for drug delivery system. The results showed that the drug release is inversely proportional to the aerogel density. Hence, the drug release rate from a silica aerogel was reduced by increasing its density. 

The first-order release kinetic model has the most goodness fit of the release data of pure drug and drug-loaded silica aerogels. However, the zero-order and Higuchi models were not suitable to describe the Ketoprofen release, particularly from silica aerogels. According to the *in-vitro* release data, a suitable relationship is found and proposed to Ketoprofen release prediction from silica aerogel with the known density.
